# Concurrence of Danish Dementia and Cataract: Insights from the Interactions of Dementia Associated Peptides with Eye Lens α-Crystallin

**DOI:** 10.1371/journal.pone.0002927

**Published:** 2008-08-13

**Authors:** Ira Surolia, Sharmistha Sinha, Debi Prasad Sarkar, P. Yadagiri Reddy, G. Bhanuprakash Reddy, Avadhesha Surolia

**Affiliations:** 1 Department of Biochemistry, South Campus, Delhi University, New Delhi, India; 2 Molecular Biophysics Unit, Indian Institute of Science, Bnaglore, India; 3 National Institute of Nutrition, Jamai-Osmania, Hyderabad, India; 4 National Institute of Immunology, Aruna Asafali Marg, New Delhi, India; University of Arkansas for Medical Sciences, United States of America

## Abstract

Familial Danish Dementia (FDD) is an autosomal disease, which is distinguished by gradual loss of vision, deafness, progressive ataxia and dementia. Cataract is the first manifestation of the disease. In this article, we demonstrate a specific correlation between the poisoning of the chaperone activity of the rat eye lens α-crystallins, loss of lens transparency in organ culture by the pathogenic form of the Danish dementia peptide, i.e. the reduced Danish dementia peptide (redADan peptide), by a combination of *ex vivo*, *in vitro*, biophysical and biochemical techniques. The interaction of redADan peptide and lens crystallins are very specific when compared with another chaperone, HSP-70, underscoring the specificity of the pathogenic form of Danish dementia peptide, redADan, for the early onset of cataract in this disease.

## Introduction

With the rise in the average age of population, neurodegenerative diseases are becoming increasingly common resulting in a greater burden on the healthcare system of most countries. Many of these perplexing disorders are known to arise from the conformational instability resulting in misfolding of an underlying protein [1], [2]. The consequence is a continuum of pathologies with typically a change in fold leading to ordered aggregation and tissue deposition. Conformational diseases arise when a constituent protein undergoes a change in size or fluctuation in shape, with resultant self-association and tissue deposition. Normally, these deposits are β-pleated sheets with distinct morphological features and, once formed, mostly remain unperturbed. Although such changes can occur with normal proteins, there is commonly an interacting genetic contribution, which may sometimes be a dominant determinant. Many of these proteins have critical roles in signaling and cell cycle control, which are perhaps responsible for their enormous impact on human health. There are at least a dozen of diseases known to arise due to protein misfolding or aggregation, which have devastating medical and social consequences [3].

Diseases such as Alzheimer's, Parkinson's and Mad cow have a number of pathological symptoms. The two familial amyloidoses, viz familial British and Danish dementias (FBD and FDD), are the two neurodegenerative disorders which are linked to different genetic defects in the same gene, BRI. Histological signatures of FDD are cerebral amyloid angiopathy, parenchymal protein deposits and neurofibrillary degeneration [4]. The 34 amino acid Danish dementia (ADan) peptide is thought to be the major cause of amyloid deposition in brains of patients suffering from FDD, whereas a 34-amino acid-long ABri peptide is believed to be the culprit of FBD. While they originate from different genetic defects in the BRI gene, ADan and ABri peptides have a number of common features. The amyloid precursor proteins in the British family (ABriPP) and in the Danish family (ADanPP) have the same length of 277 amino acids. Also, both the peptides have the same sequence length and share identical N-terminal amino acid sequence (the first 22 residues), which suggests that these molecules are generated by the same proteolytic mechanism [4]. Earlier reports on ADan peptides have shown that both the oxidized (oxAdan; Cys5 and Cys22 linked by an intramolecular disulfide bond) and the reduced (redAdan; no intramolecular disulfide bond present) forms of the peptide are incompetent in producing fibers at pH 7.0 [5]. However, we demonstrated that, the morphology of the aggregates formed depends greatly on the form of the peptide (viz. the oxidized *versus* the reduced peptide) [6]. We also reported that the neurotoxicity of the reduced form is greater than that of the oxidized form [6], [7].

FDD is also known as heredo-oto ophthalmo-encephlopathy (HOOE). HOOE/FDD is a dominantly inherited syndrome clinically characterized by a gradual loss of vision, deafness, progressive ataxia and dementia [8], [9]. Visual loss in FDD is caused by posterior subcapsular cataract and retinal neovascularization. Cataracts seem to be the earliest manifestation of this disease, starting at the age of 20 followed by hearing impairment developing at the age of 40. Cerebellar ataxia then follows and consequently symptoms of paranoid psychosis and dementia are observed [10]. Early-onset of cataract is also seen in Alzheimer's and Down syndrome patients [11].

Cataract, characterized by cloudiness or opafication of the eye lens, is the leading cause of blindness all over the world. Crystallins are the major structural proteins in the lens accounting for up to 90% of total soluble protein. There are three distinct families of crystallins: α-, β- and γ-crystallins, whose structure, stability and short-range interactions are thought to contribute to the lens transparency [12]. The human lens is also susceptible to age-related degenerative changes such as accumulation of insoluble proteins and oxidative damage and hence senile cataracts are the most common form of the cataract [12], [13]. α-Crystallin, a member of the small heat shock protein family, constitutes a major portion of the eye lens cytoplasm. It constitutes up to 50% of the total protein [14], [15], [16], [17]. α-Crystallin monomer has Mr. of 20,000. In humans, lenticular α-crystallin exists as a hetero-oligomer of approximately Mr. 800,000 with two subunits, αA and αB occurring in a stoichiometry of 3∶1 [16]. αA-Crystallin appears to be largely lens-specific, whereas αB-crystallin is also expressed in other tissues such as heart, skeletal muscle, kidney, and brain. Increased levels of αB-crystallin have been observed in many neurodegenerative disorders, tumors and diabetic conditions [18], [19]. Both of these proteins are known for their chaperone activity as evident from suppression of protein aggregation. Thus they presumably protect other lens proteins from the adverse effects of heat, chemicals, and UV light irradiation. It is widely acknowledged that with aging eye lens proteins undergo various posttranslational modifications, most of which lead to aggregation and this process is further accelerated due to various physiological, environmental and genetic factors that predispose lens to cataract formation [12], [13]. Hence, in addition to providing refractive properties to the eye lens, α-crystallins are instrumental in maintaining transparency of the lens with their chaperone-like activity [12], [20], [21]. Recently it has been reported that some low molecular weight peptides found in aged and cataractous lens bind and reduce the chaperone activity of α-crystallin [22].

This article addresses the relationship between cataract and Danish dementia, which is unique among amyloid disorders as its earliest manifestation involves cataract. As α-crystallin plays a pivotal role in maintaining the lens transparency and its damage is reported to play a major casual role in cataracts [23], [24] we have probed the interaction of the Danish dementia and some other dementia associated peptides with the lens α-crystallin *in vitro* and *ex vivo*. Our studies demonstrate that ADan reduced peptide (redADan) has both exquisite specificity and ability among several amyliodogenic peptides to compromise the chaperonic function of α-crystallin as well disposing lens in organ culture to cataractogenesis all of which help explain the high incidence of the occurrence of cataract in Danish dementia.

## Results

### Changes in lens morphology upon interaction of the ADan, ABri and Aβ peptides:

**Figure 1:**
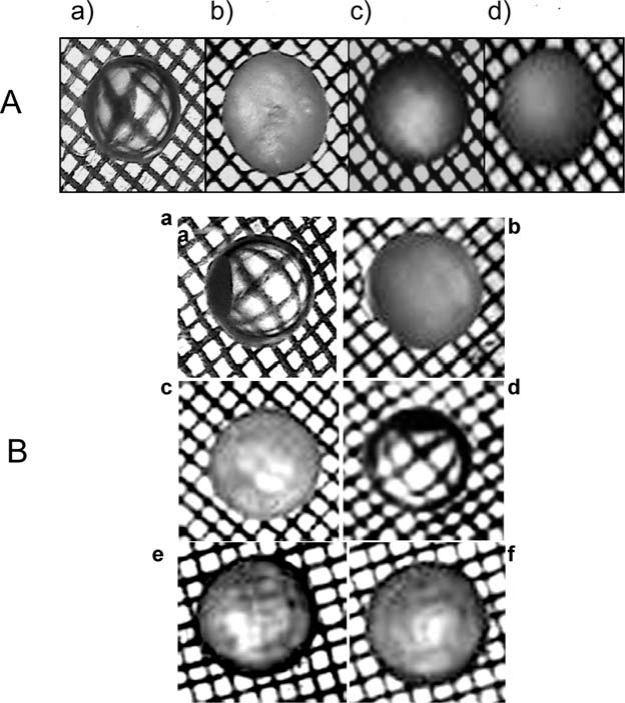
Opafication of lenses from adult Wistar rats cultured in TC-199 medium with and without dementia related peptides. (A) Opafication of rat lenses cultured with 0 µM (a), 5 µM (b), 10 µM (c) & 25 µM (d) redADan peptide. TC-199 medium was changed every 24 h. At 96 h the lenses were evaluated for their opafication as described in the method. (B). In each case 10 µM of the peptide with a rat lens in the culture medium was used and the peptide containing medium was changed every 24 h. At 96 h the lenses were processed for evaluating their opafication as described in the text. a: Control Lens; b: redAdan; c: oxAdan; d: redABri; e: oxABri and f: Aβ-40 peptide.

Initial studies with redADan showed that lenses incubated for four consecutive days with 10 µM redADan peptide concentration became cloudy and lost transparency completely (Figure 1a). Hence, the effect of the various dementia-associated peptides on the opafication of lenses was studied at 10 µM concentration. However, at this concentration oxADan peptide or redABri peptides did not affect lens transparency to any appreciable extent [Figure 1b]. Also lenses incubated with 10 µM of oxABri or Aβ-40 peptide exhibited a partial opafication only. This opafication is close to 50% of that observed with the redADan peptide [Figure 1].

### Effect of the ADan, ABri and Aβ peptides on the chaperone activity of α-crystallins:

**Figure 2:**
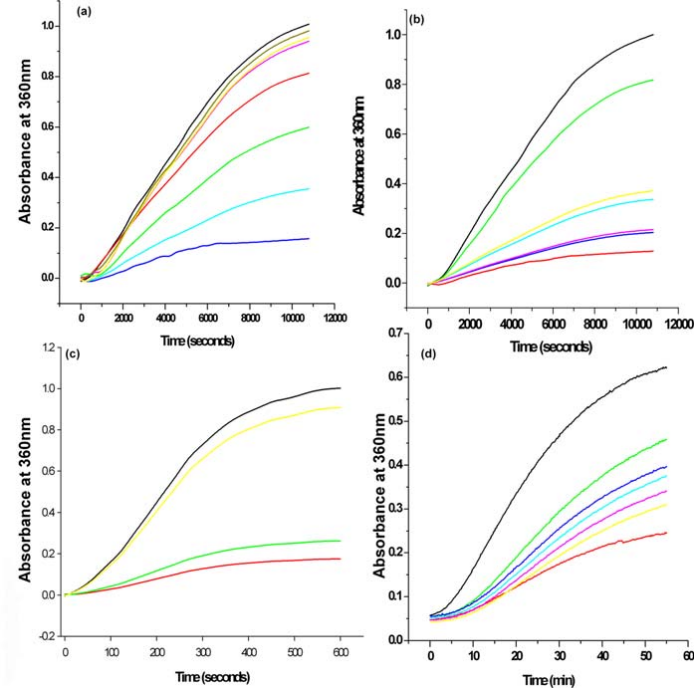
(a) Chaperone activity of αA-crystallin as assessed by the suppression of heat induced aggregation of citrate synthase in the presence of different concentrations of redADan peptides. Citrate Synthase (0.05 mg/ml) was incubated in the absence (*black*) or presence of 10 µM α-crystallin (*dark blue*), or lenses treated with 2.5 µM redADan (*light blue*), 5 µM redADan (*green*), 10 µM redADan (*red*), 20 µM redADan (*pink*), 50 µM redADan (*yellow*) and 100 µM redADan (*brown*) (b) Chaperone activity of αA-crystallin (10 µM) as assessed by the suppression of heat induced aggregation of citrate synthase (0.05 mg/ml) at 43°C in the presence of dementia related peptides (*dark blue*) redAbri, (*pink*) oxADan, (*light blue*) oxAbri, (*yellow*) Aβ-40, (*green*) redADan (10 µM of each of the peptides). (c) Chaperone activity of αA-crystallin as assessed by the suppression of DTT induced aggregation of insulin. Insulin was incubated in the absence (*black*) and presence of 10 µM α-crystallin (*red*), oxADan (*green*) and redADan (*yellow*) peptides (Insulin, 0.2 mg/ml; DTT, 20 mM at pH 7.2) (d) Chaperone activity of αL-crystallin as assessed by the suppression of heat induced aggregation of citrate synthase at 43°C. Citrate synthase, 0.05 mg/ml, was then incubated in the absence (*black*) or presence of 10 µM α-crystallin isolated from control lenses (*red*), or lenses treated with oxAdan (*yellow*), redABri (*pink*), Aβ−40 peptide (*light blue*), oxABri (*dark blue*) and redAdan (*light green*).

**Figure 3:**
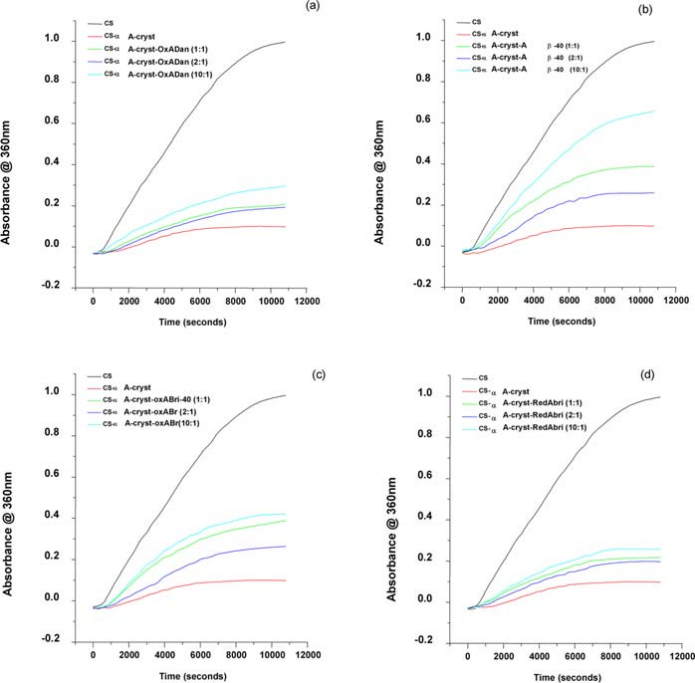
Chaperone activity of αA-crystallin (10 µM) as assessed by the suppression of heat induced aggregation of citrate synthase (0.05 mg/ml) at 43°C in the presence of different concentrations of (a) oxADan (b) Aβ-40 peptide (c) oxABri(d) redABri. [black: citrate synthase; red: citrate Synthase + αA-crystallin; dark blue: citrate Synthase + Dememtia peptide : αA-crystallin (1∶1); green: citrate Synthase + Dememtia peptide: αA-crystallin (2∶1); light blue: citrate Synthase + Dememtia peptide: αA-crystallin (10∶1)].

The poisoning of chaperone activity of crystallins in the presence of these peptides was monitored by the heat induced citrate synthase aggregation and DTT-induced insulin aggregation profiles (Figure 2) [19], [25]. It was observed that the peptides were poisoning to a variable extent the chaperone activities of the α-crystallins, αA and αB. While in the case of redADan the poisoning was most remarkable, the other peptides exhibited less poisoning of the chaperone activity. Thus at equimolar concentrations of ADan there was almost 80% poisoning of the chaperone activity of αA-crystallin. About 50% and 30% poisoning was observed when redADan: αA-crystallin were used in the ratios of 1∶2 and 1∶4 respectively (Figure 2a). Moreover, almost 100% poisoning of αA-crystallin was obtained when the ratio of redADan used was 5∶1 and 10∶1. On the other hand, Aβ-40 peptide and oxABri accounted for only ∼30% of the poisoning at equimolar concentration of αA-crystallin. Interestingly, only 10% loss of the chaperone activity of αA-crystallin, at equimolar concentrations of redABri and oxADan peptides was observed (Figure 2b). Similar results were observed with insulin aggregation assays for αA- crystallin (Figure 2c). The effect of different concentrations of the dementia peptides on the chaperone activity of αA-crystallin was also studied. Increasing the concentrations of oxABri, redABri and oxADan to 10 times the αA-crystallin concentration did not show any marked difference in the poisoning of the chaperone activity of the crystallin compared to the equimolar situation for oxABri, redABri and oxADan. However, under similar conditions the chaperone activity was compromised to the extent of 60 % by the Aβ-40 peptide (Figure 3).

The chaperone activity of αB-crystallin at an equimolar concentration of redADan was compromised slightly more (∼90%) over that observed for αA-counterpart which is not surprising in view of the previous observations about the relative activities of these crystallin isoforms ( Figure S1). Similar results were observed with insulin aggregation assays for αA-crystallin (Figure 2c). Due to the relative abundance of αA form of crystallin in both the human & rat eye lenses, unless otherwise stated, most of our studies reported here were carried out with αA-crystallin.

### Chaperone activity of the α-crystallin isolated from rat lenses incubated with the peptides:

While, αL-crystallin isolated from the rat lenses treated with redADan showed significantly (48%) decreased chaperone activity as compared to αL-crystallin isolated from untreated lenses in citrate synthase aggregation assay. In comparison, the reduction in chaperone activity of αL-crystallin isolated from lenses treated with Aβ-40 peptide, oxABri, redABri and oxAdan was 39%, 36%, 31% and 27% respectively.

### Changes in lens protein content upon interaction of the ADan, ABri and Aβ peptides:

Generally lens opafication or cataract is associated with insolubilization of otherwise soluble lens proteins. Therefore, estimation of the protein content in both the soluble and insoluble fractions of the lens extract (homogenate) was done. Insolublization of soluble protein in the redADan treated lenses was remarkably high as the percent soluble fraction was reduced to 48% compared to 81% in control lenses [Table 1]. The reduction in soluble protein content in the soluble fraction of lenses treated with oxADan, redABri, Aβ-40 and oxABri was 69%, 70% 67% and 64% respectively. Thus the lenses treated with the redADan peptide yielded the least amount of soluble protein in the soluble fraction (Table 1).

**Table 1:** Effect of peptides on lens protein content in rat lens

Parameter	Control	redAdan	oxAdan	redABri	oxABri	Aβ (1–42)
Total protein (mg/g lens)	518	483	485	496	456	464
Soluble protein (mg/g lens)	421	232	338	346	292	310
Insoluble protein (mg/g lens)	95	240	135	141	150	145
% Soluble protein a	81	48	70	70	64	67

a Percentage soluble protein is calculated in relation to the amount of total protein

### Determination of dissociation constants for the interactions of α-crystallins with ADan, ABri and Aβ peptides:

**Figure 4:**
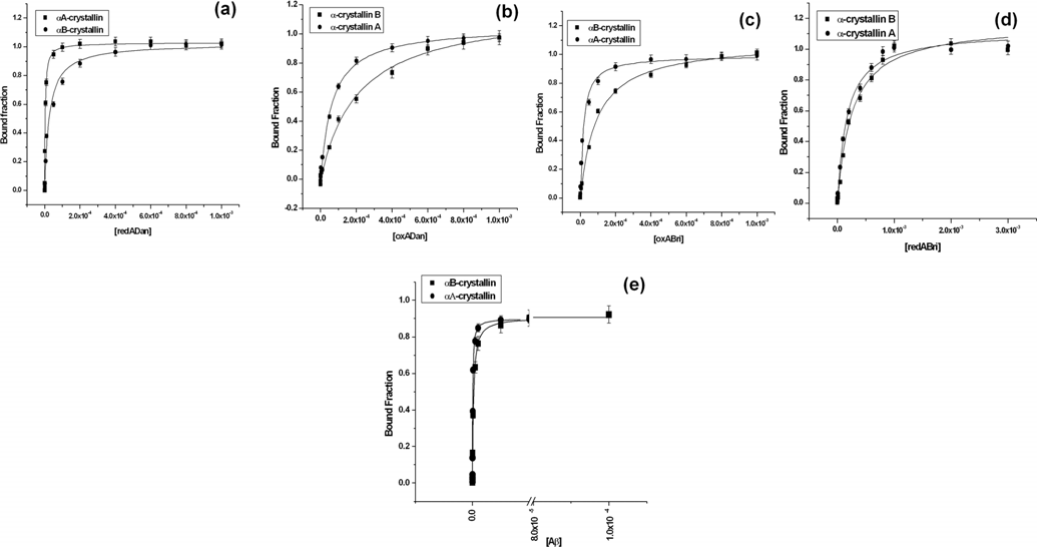
Spectrophotometric determination of the dissociation constant for the interaction of α-crystallin (A and B) with (a) redADan (b)oxADan (c)oxABri (d) redABri (e)Aβ-40 peptide.

The dissociation constants (K_d_) for the interaction of the five dementia associated peptides with α−crystallins presented very interesting results. While Aβ-40 peptide demonstrated nanomolar range dissociation constant, the K_d_ values for the interaction of the other peptides with both α-crystallins were in the micromolar range. Our results corroborate well with an earlier report on the binding of α-crystallins to Aβ-peptide [11]. Isothermal Titration Calorimetric (ITC) binding studies were also done with redADan peptide and αA-crystallin. The value of the K_d_ obtained from the nonlinear least squares fit of the data is ∼1.7 µM (Figure S2), which is in close proximity to the values obtained spectroscopically for redADan αA-crystallin association viz. 2.7 µM. However, the ITC fit obtained has a high χ^2^ value perhaps due to the aggregation of the peptides in solution at the concentrations required for ITC studies. Hence, ITC was not used for further studies. The dissociation constants obtained using the spectrophotometric assay of these peptides binding to both αA-crystallin and αB-crystallin are presented in Figure 4 and the corresponding K_d_ values are listed in Table 2. The K_d_ shows the following trend:

**Table 2:** Dissociation constants (K_d_) of α-crystallins with peptides at 25°C

Peptides	αA-crystallin	αB-crystallin
Aβ-40	56.9×10^−9^±4.2×10^−9^ M	184.8×10^−9^±14.7×10^−9^ M
RedADan	2.7×10^−6^±1.82×10^−7^ M	4.1×10^−6^±1.94×10^−6^ M
OxADan	6.50×10^−5^±5.43×10^−6^ M	2.12×10^−4^±1.952×10^−5^ M
RedABri	1.7×10^−4^±1.6×10^−5^ M	2.5×10^−4^±3.1×10^−5^ M
OxABri	17.5×10^−6^±1.75×10^−7^ M	92.2×10^−6^±6.17×10^−6^ M

Aβ−40 peptide >redADan>oxABri>redABri∼oxADan.

However, the trend in the loss of chaperone activity of α-crystallin does not parallel the trend of binding affinities exhibited by these peptides for this chaperone. This suggests that the affinity of interaction (viz the dissociation constant) between the crystallins & the dementia peptides do not correspond strictly with their pathogenic effects in the organ culture, pointing towards specificity in catractogenesis caused by redADan. Significantly, redADan also exhibits greatest potency in inhibiting chaperone activity of both αA and αB-crystallin.

### ANS binding studies

**Figure 5:**
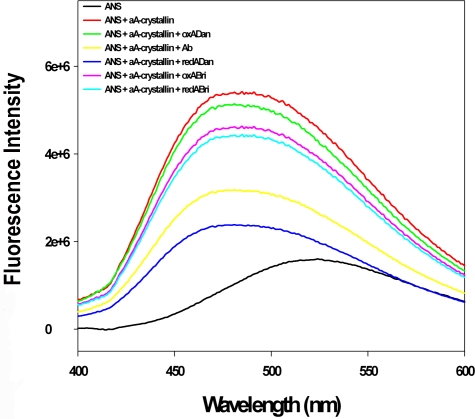
Fluorescence emission spectra of ANS in the presence and absence of αA-crystallin and the various dementia associated peptides. The crystallin and the peptide concentration used were 20 µM and 50 µM respectively. The samples were excited at 370 nm and emission scan was obtained from 400 to 600 nm.

Further, to probe the nature of interaction between α-crystallin and the dementia associated peptides, 8-anilino-1-napthalenesulfonic acid (ANS) was used to measure the extent of hydrophobic surface of αA-crystallin in the presence and absence of redADan and the other peptides used in this study. While a considerable decrease in the intensity at 470 nm for ANS complexed to αA-Crystallin occurs in the presence of redADan peptide (80%), other dementia associated peptides compromised in a feeble manner the fluorescence of ANS complexed to this chaperone. Thus, in the presence of Aβ-40 the fluorescence intensity was diminished by 55%, while, redABri and oxAbri account for ∼33% loss in the fluorescence intensity. For oxADan the decrease in ANS fluorescence was only 11% (Figure 5).

## Discussions

Previous studies from our laboratory have shown that while oxADan peptide give rise to fibrils that are stable and birefringent, redADan yields only dome shaped non-birefringent intermediates [6]. Nevertheless, it is the early oligomers of redADan that are much more effective in neuropathogenesis as assayed by MTT assay at lower concentrations than those of oxADan counterparts [6], [7]. In ABri peptides, oxABri not only leads to fibril formation but also constitutes the toxic form unlike its reduced counterpart, redABri. Early age lens cataract is a significant pathological signature of Danish dementia [8]. This article sheds light on the issue of the relationship between Danish dementia and eye lens cataract. For this purpose we initially probed the interaction of α-crystallin with ADan, ABri and Aβ-40 peptides employing a number of *in vitro* and *ex vivo* techniques. Results of our study show that out of the five genres of peptides examined here, the redADan is most instrumental in rendering rat lenses opaque. Aβ-peptide and oxABri peptides show in comparison a considerably reduced extent of lens opafication. RedABri and oxADan on the other hand show a remarkably reduced loss of transparency of the lenses. Our studies thus indicate as to why redADan (the pathogenic form of Danish dementia peptide) displays the striking hallmark viz. cataract, often intimately linked with the earliest symptoms of Danish dementia [5], [6], [26].

OxADan and redABri peptides show relatively low binding constants to α-crystallin while compromising its chaperone activity marginally only. Moreover, they were also poor in inducing opafication of lenses in organ culture. However, the reason as to why oxABri peptide also showed inhibition of the chaperone activity of α-crystallin is somewhat unclear. One of the reasons may be related to the fact that α-crystallin has exposed hydrophobic sites which may provide a surface for a non-specific association of oxABri which is known to aggregate rapidly. Thus the effect of oxABri may be a secondary event which may be related to nonspecific factors such as cross-linking and consequent aggregation of the lens proteins. This indeed appears to be the case in the light of the data discussed below.

Since inhibition of the chaperone activity of lens crystallins is primarily shown to be responsible for the ease and specificity with which redADan manifests appearance of cataract in lens organ culture, we also studied the effect of redADan on another chaperone. For this purpose the efficacy of redADan and other dementia related peptides on the chaperone function of human HSP-70 was examined [27]. None of the dementia peptides compromise in any significant manner the activity of HSP-70 (Figure S3). This is significant as it shows that the interaction of the redADan peptide with the α-crystallin and the subsequent loss of the chaperone activity of the latter are very specific which underlie cataract, one of the hallmarks of the pathogenesis observed in Danish dementia.

As the structure of α-crystallin is not available it becomes a bit difficult to point out the exact sites in it that may be interacting with these dementia-associated peptides. The exposure of hydrophobic surface on α-crystallins is intrinsic to their chaperone functions. Hence, the exposure of hydrophobic surface on the free αA-crystallin and the αA-crystallin complexed to the dementia associated peptides was probed with ANS. OxADan, redABri and oxABri show 10–35% reduction in ANS fluorescence. Aβ-40 peptide exhibits 45–55% reduction in fluorescence of ANS complexed to αΑ-crystallin as compared to 80% loss observed in the presence of redADan. The fact that oxABri compromises in a feeble manner the fluorescence of ANS-αA-crystallin complex lends support to our interpretation in general about the non-specificity of the affect of oxABri on lens opafication. Hence, the poisoning of the chaperone activity of α cystallins by redADan and Aβ-40 peptide and their strong affinities for binding to crystallins are consistent with the observed loss of the exposed hydrophobic areas on its surface. This in turn is consistent with the greater potencies of redADan and Aβ-40 peptide in rendering the lenses opaque as demonstrated in these studies.

In conclusion, our studies show a remarkable correlation between the ability of redADan peptide to incapacitate the chaperone activity of α-crystallin *in vitro* with its ability to cause opafication of eye lenses *ex-vivo*. Other dementia related peptide despite their established role in amyloid diseases, exhibit relatively poor inhibition of the chaperone function of eye lens crystallins as well as considerably reduced opafication of lenses in organ culture as compared to the redADan explaining as to why in FDD alone among the amyloid diseases an early onset of cataract is observed. These results provide a mechanistic basis for the dominant HOOE/FDD syndrome viz. the poisoning of chaperone-like function of α-crystallins. Studies that report the role of α -crystallin in other neurodegenerative conditions support this possibility. For example, Aβ-40 peptide is known to be expressed in rodent and monkey lens and oxidative stress has been shown to increase the production of Aβ precursor protein and Aβ-40 peptide [28]. It was demonstrated that Aβ peptide has toxic effects on lens epithelial cells [28]. Aβ peptide was also shown to interact with αB-crystallin [29], [30].

## Materials and Methods

### Materials

Oxidized and reduced Danish dementia peptides (oxADan and redADan), oxidized and reduced British dementia peptides (oxABri and redABri), Aβ-40 peptide were bought from Bachem AG (Switzerland) and purified using a C-18 HPLC column in on a WATERS HPLC. The purity and mass of the peptides were assessed by MALDI-TOF mass spectrometer (Bruker Daltonics) which were found to correspond to their expected masses of 4046.74 Da, 4044.52 Da, 3937.81 Da, 3939.88 Da and 4330.01 Da for redADan, oxADan, redABri, oxABri and Aβ-40 peptides respectively (Figure S4). The peptides were subjected to a HFIP treatment as mentioned in [31] with a slight modification. Briefly, the peptides were dissolved in HFIP (1, 1, 1, 3, 3, 3-hexafluoro-2-propanol) and sonicated in a water bath for 1 hour, followed by vortexing for 15 minutes. The solution was left in HFIP overnight. The resulting solution was lyophilized and taken for fibrilization. The lyophilized peptides thus obtained were dissolved in a minimum volume of DMSO (dimethyl sulphoxide) under a flow of liquid nitrogen. These were the stocks. In this way peptides could be stored in DMSO for a period of almost a month without any aggregation. Human HSP-70 was purchased from Sigma.

### Purification of Recombinant αA- and αB-crystallins:

Expression vectors (pET23d) of human αA- and αB-crystallin were a generous gift from Dr. J. Mark Petrash, Department of Opthalmology and Visual Sciences (Washington University, St. Louis, MO, USA). Human αA- and αB-crystallins were expressed in bacterial (BL21) cells containing expression vectors (pET23d). Proteins from 1-liter cultures were extracted and purified according to the procedures described previously [25]. Briefly, αA- and αB-crystallins were overexpressed in *Escherichia coli* BL21 cells containing the respective vectors by isopropyl 1-thio-β-D-galactopyranoside induction and purified using MonoQ anion exchange and Sephacryl S-300 gel filtration columns. The purity/homogeneity of αA- and αB- crystallins was found to be 99% as analyzed by SDS-PAGE. Concentrations of αA and αB crystallins were calculated using molar extinction coefficients, (ε280), of 16,500 and 19,000 M^−1^ cm^−1^, respectively.

### Chaperone Activity Assay

The chaperone activities of αA-crystallin and αB-crystallin were assessed by measuring each one's ability to prevent the heat induced aggregation of citrate synthase at 43°C and DTT-induced aggregation of insulin at 25°C [25]. Apparent scattering of the solution at 360 nm due to heat induced aggregation of citrate synthase at 43°C at pH 7.9 (HEPES-KOH buffer), was monitored as a function of time in the absence and presence of αA- or αB-crystallins using a Jasco UV-visible spectrophotometer. The amount of α-crystallins used was 5 µg/µL and the total volume of the assay was 100 µL in 1 cm path length quartz cuvette. For insulin aggregation assay the reaction was initiated by addition of 20 mM DTT in a solution containing 5 µg /ml αA-crystallin or αB-crystallin and 0.2 mg/ml insulin at pH 7.2 in phosphate buffer. The total volume of the reaction was kept to 100 µl. Reactions were carried out at room temperature.

Assay for the chaperone activity with the human heat shock protein HSP-70 was carried out as described in [27]. Its chaperone activity was assayed by its ability to prevent thermal aggregation of glutamate dehydrogenase (GDH). The assay mix comprised of 20 mM Tris buffer pH 7.4, 100 mM sodium chloride, 0.2 mg/ml GDH and 0.2 mg/ml human HSP. The assay was carried out at 48°C using a peltier controlled cell and was monitored by measuring the light scattering at 360 nm in a Jasco sphectrophotometer. GDH was used in the assay to compare the results of this study with those obtained in [27].

### Determination of the binding constants of α-crystallins to peptides

The binding of αA-crystallin and αB-crystallin with Aβ-40, ADan and ABri peptides were assessed spectrophotometrically using modification of the method detailed in [11]. In the absence of crystallins all the peptides upon incubation at 25°C for 12 h are found in the sediment upon centrifugation at 21,000 g for 30 min. Crystallins keep them in solution under these conditions. Hence, the ability of crystallins to keep the peptides in solution was used to measure the affinity of their interactions with the peptides. Briefly, 2 µM of α-crystallins were incubated overnight (12 h) at 25°C with different concentrations of the peptides spanning nanomolar to millimolar concentration range in HEPES buffer (pH 7.8). In another set of experiments α-crystallins were omitted and only the peptides alone were incubated at these concentrations overnight at 25°C. Samples from both the sets were centrifuged at 21,000 g for 30 min and the supernatant and the precipitate were separated out. The optical density of the supernatant fraction was taken at 215 nm. The difference in the absorbance before and after centrifugation gives the amount of peptide aggregated. The difference in the amount of peptide aggregated in the samples with and without the crystallins gives the measure of the association of crystallins with the peptides. The data were fitted to the following equation:
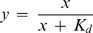
(1)Where, y is the difference in the amount of peptide associated with α-crystallin and x is the concentration of the free peptide.

### Lens organ culture:

Eyes were enucleated from 4 months old Wistar rats, obtained from National Center for Laboratory Animal Services, National Institute of Nutrition, Hyderabad immediately after sacrifice by cervical dislocation. Animal care and protocols were in accordance with and approved by Institutional Animal Ethics Committee. Lenses were dissected from the eyes by anterior approach. Each isolated lens was incubated in 2 ml of modified of TC-199 (Sigma) medium with antibiotics. Osmolarity of the medium was adjusted to 295 milli osmomoles using an osmometer (Micro Osmometer; Model-210, Fiske Associates, Norwood, MA, USA). Lenses were cultured as described previously [32], [33]. Lenses were incubated at 37°C under 95% air and 5% CO_2_ in the absence or presence of the various dementia associated peptides dissolved in DMSO for a period of 96 h. All the reagents/chemicals used for lens organ culture were filtered through 0.2 µm Millipore disc filters. Final concentration of a given dementia associated peptides in the culture medium was kept at 10 µM. Medium was changed every 24 h. At the end of incubation, lenses were kept on a grid (12/cm) for assessing the transparency by placing them above a light source.

### Extraction of proteins from the treated lenses

Lenses were homogenized in 10 vol. of 0.05 M Tris buffer (pH 8.0), containing 0.1 M NaCl and 0.02% sodium azide. The homogenate was centrifuged at 15 000 *g* for 30 min at 4°C and the resulting supernatant was collected (soluble fraction). The pellet was washed thrice and constituted in the same buffer (insoluble fraction). Protein content in soluble and insoluble fractions was estimated by Lowry's method.

### Purification and evaluation of the chaperone activity of α -crystallin (αL-crystallin) isolated from peptide treated lenses

α-Crystallins from the soluble fraction of the lens extract described above (αL-crystallin) were purified by gel filtration on a Sephacryl S-300 column ( 90 ×0.62 cm) as described previously [19]. Chaperone activity of the α L-crystallin thus isolated was assayed using its ability to prevent aggregation of citrate synthase undergoing heat inactivation at 43°C as described above.

### ANS binding studies

ANS (1-anilino-8-naphthalene sulfonate) binding experiments were executed with both the free α A-crystallin and its complexes with the various dementia associated peptides. All the experiments were done in a Jobin Yvon Horiba fluorometer (Jobin Yvon Spex, Cedex, France) in a 1-cm path length quartz water- jacketed cell using αA-crystallin and dementia associated peptides at concentrations of 20 µM and 50 µM respectively. The samples were excited at 370 nm and emission scan was obtained from 400 to 600 nm.

## Supporting Information

Figure S1 αB-crystallin demonstrated similar poisoning of chaperone activity in the presence of the peptides as αA-crystallin. The amount of αB-crystallin used was 10 µM. (a) Different concentration ration of redADan peptide and αB-crystallin is used and (b) All the dementia peptides are used (concentration of the peptides 10 µM).
(1.40 MB TIF)

Figure S2 Isothermal Calorimetric Curve of the titration of 20 µM of αA-crystallin assuming its relative molecular weight Mr ∼20,000 with 250 µM of redADan peptide. (Upper panel) Raw data of heat evolved during the titration. The first injection was executed by injection of 2 µL of redADan solution in the cell. The later injections were of 8 µL. (lower panel) Least squares fit of the data in the upper panel for the determination of the thermodynamic parameters of interaction. The value of K_b_ obtained from nonlinear least squares minimization method is 0.6×10^6^.ITC was performed by using VPITC calorimeter from Microcal Inc. (Northampton, MA). αA-crystallin (∼Mr 20,000 ), 20 µM in 1.5 ml 5 mM Hepes buffer (pH 7.8), was titrated with 8 µl of redADan solution (250 µM) at an interval of 3 min using a syringe rotating at 310 rpm. The data so obtained were fitted via nonlinear least squares minimization method to determine binding stoichiometry (n), binding constant (K_b_), and change in enthalpy of binding (delta H) using Origin software (Microcal). The experimental conditions ensured that c was 12, where c = K_b_M_t_ (0) and M_t_(0) is the initial macromolecular concentration. The value of the binding constant (K_b_) was used to compare the value obtained spectroscopically.
(1.22 MB TIF)

Figure S3 Chaperone activity of *Pf*HSP-70 (0.2 mg/ml) as assessed by the suppression of heat induced aggregation of GDH at 48°C in the presence of different concentrations of redADan peptides (GDH (0.2 mg/ml)).
(0.89 MB TIF)

Figure S4 The MALDI mass spectrum of the HPLC purified dementia peptides. (a) redADan (b) oxADan peptides. (c) redABri (d) oxABri peptides and (e) Aβ-40 peptides. The disaggregated peptide solution were dissolved in water and after spinning, 0.5 µL of this sample was spotted on MALDI plate followed by 0.5 µL of alpha-cyano-4-hydroxycinnamic acid matrix (10 mg/mL in 50% acetonitrile, 0.1% TFA). After drying the plate was inserted in the voyager. All the spectra were collected in the positive ionization mode.
(1.25 MB TIF)
