# Supplementary figures and images for: Concurrence of Danish Dementia and Cataract: Insights from the Interactions of Dementia Associated Peptides with Eye Lens α-Crystallin
Source: PLoS One. 2008 Aug 13;3(8):e2927. doi: 10.1371/journal.pone.0002927 (PMC2488398; doi:10.1371/journal.pone.0002927)

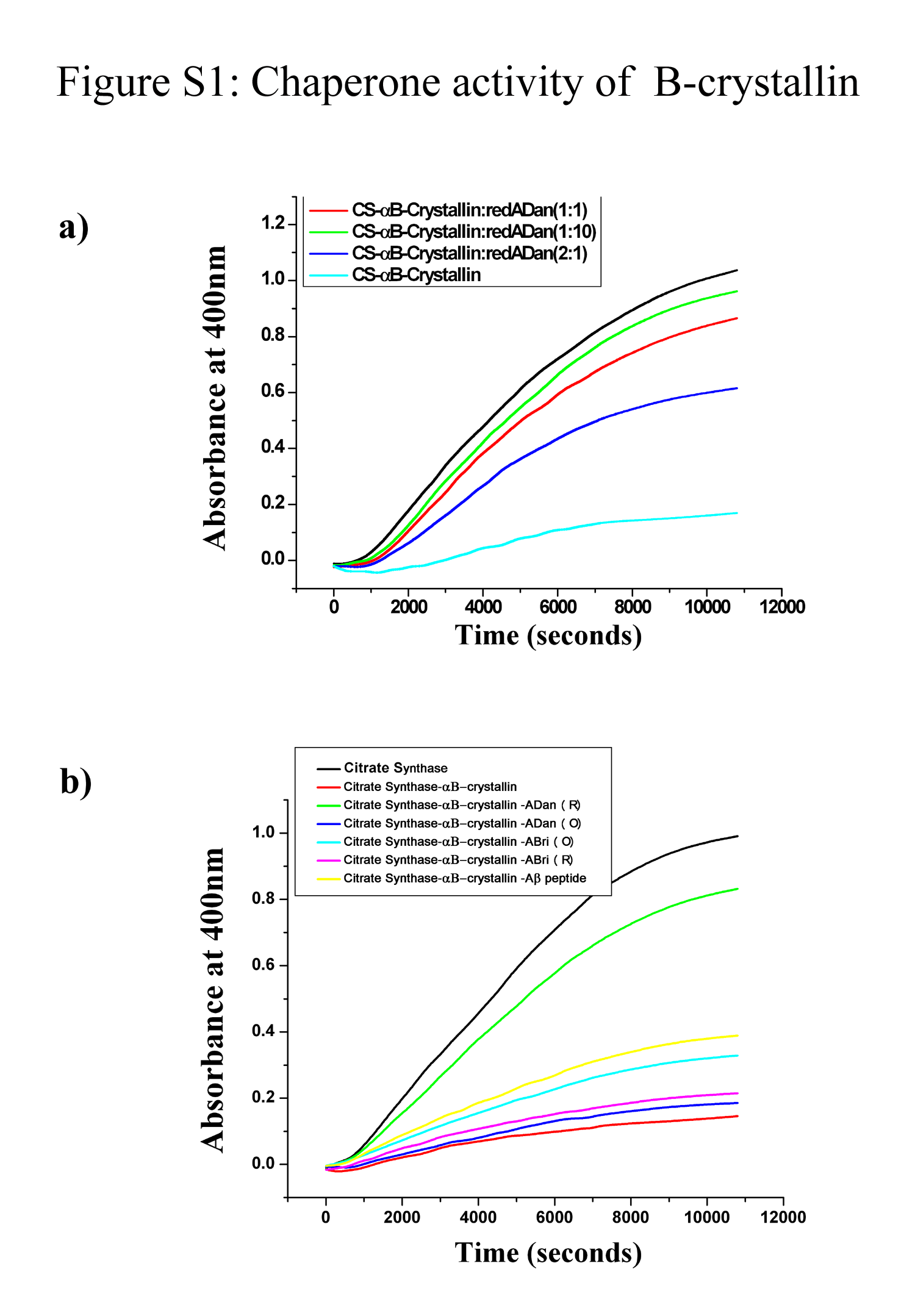

Supplement: Figure S1 — αB-crystallin demonstrated similar poisoning of chaperone activity in the presence of the peptides as αA-crystallin. The amount of αB-crystallin used was 10 µM. (a) Different concentration ration of redADan peptide and αB-crystallin is used and (b) All the dementia peptides are used (concentration of the peptides 10 µM). (1.40 MB TIF) [file pone.0002927.s001.tif]

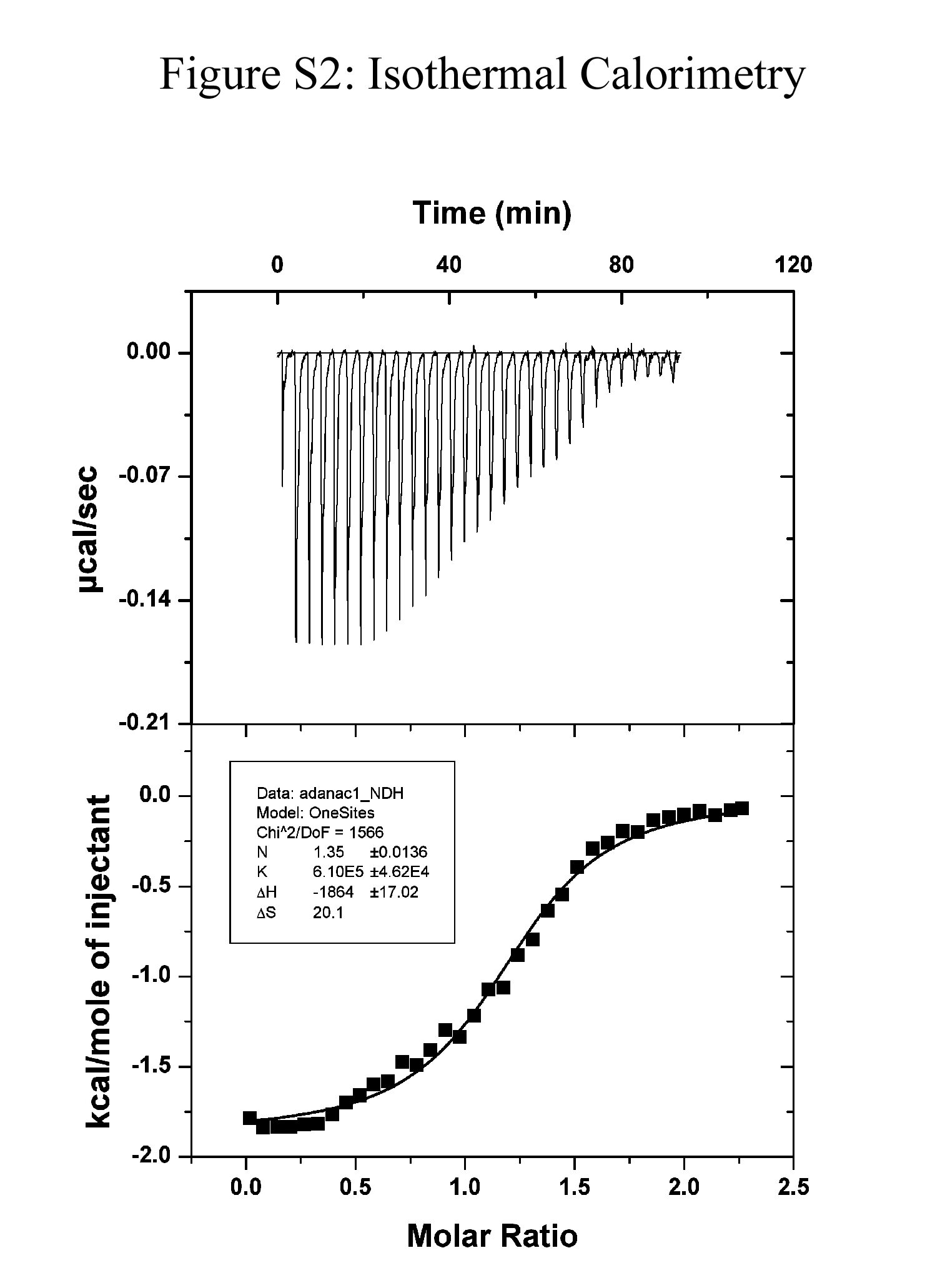

Supplement: Figure S2 — Isothermal Calorimetric Curve of the titration of 20 µM of αA-crystallin assuming its relative molecular weight Mr ∼20,000 with 250 µM of redADan peptide. (Upper panel) Raw data of heat evolved during the titration. The first injection was executed by injection of 2 µL of redADan solution in the cell. The later injections were of 8 µL. (lower panel) Least squares fit of the data in the upper panel for the determination of the thermodynamic parameters of interaction. The value of Kb obtained from nonlinear least squares minimization method is 0.6×10^6^.ITC was performed by using VPITC calorimeter from Microcal Inc. (Northampton, MA). αA-crystallin (∼Mr 20,000 ), 20 µM in 1.5 ml 5 mM Hepes buffer (pH 7.8), was titrated with 8 µl of redADan solution (250 µM) at an interval of 3 min using a syringe rotating at 310 rpm. The data so obtained were fitted via nonlinear least squares minimization method to determine binding stoichiometry (n), binding constant (Kb), and change in enthalpy of binding (delta H) using Origin software (Microcal). The experimental conditions ensured that c was 12, where c = KbMt (0) and Mt(0) is the initial macromolecular concentration. The value of the binding constant (Kb) was used to compare the value obtained spectroscopically. (1.22 MB TIF) [file pone.0002927.s002.tif]

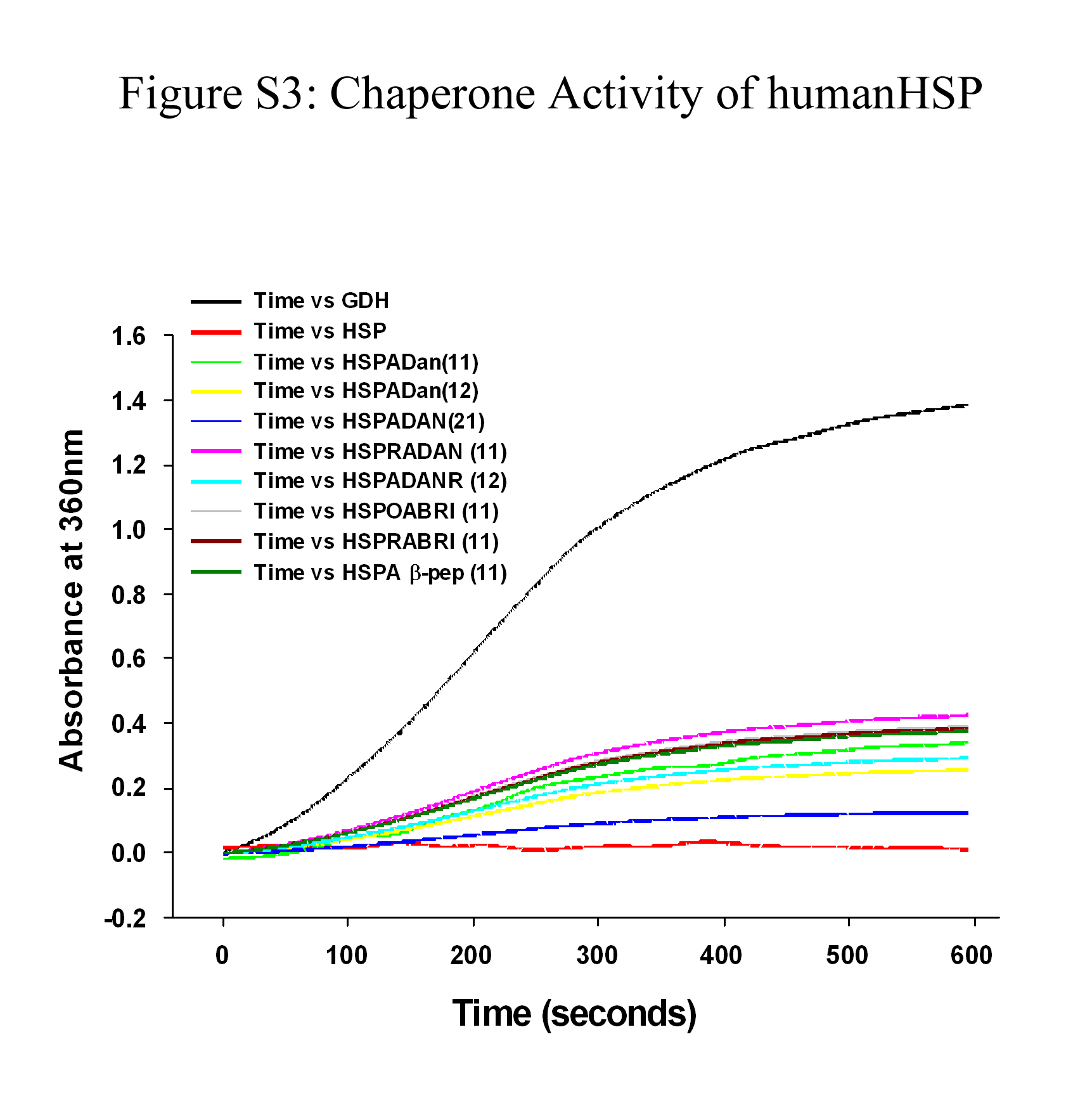

Supplement: Figure S3 — Chaperone activity of PfHSP-70 (0.2 mg/ml) as assessed by the suppression of heat induced aggregation of GDH at 48°C in the presence of different concentrations of redADan peptides (GDH (0.2 mg/ml)). (0.89 MB TIF) [file pone.0002927.s003.tif]

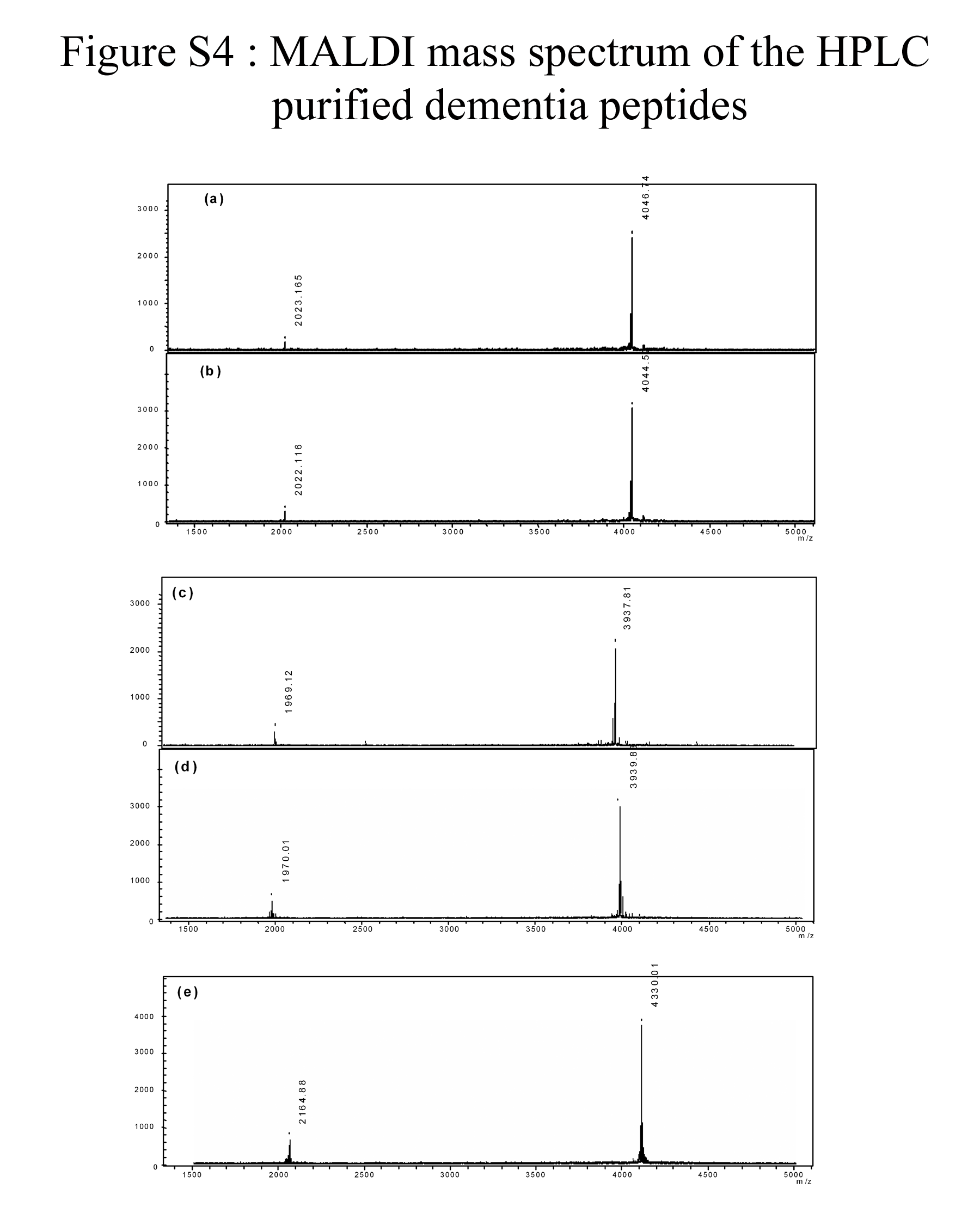

Supplement: Figure S4 — The MALDI mass spectrum of the HPLC purified dementia peptides. (a) redADan (b) oxADan peptides. (c) redABri (d) oxABri peptides and (e) Aβ-40 peptides. The disaggregated peptide solution were dissolved in water and after spinning, 0.5 µL of this sample was spotted on MALDI plate followed by 0.5 µL of alpha-cyano-4-hydroxycinnamic acid matrix (10 mg/mL in 50% acetonitrile, 0.1% TFA). After drying the plate was inserted in the voyager. All the spectra were collected in the positive ionization mode. (1.25 MB TIF) [file pone.0002927.s004.tif]
